# An Experimental Theory Concerning the Formation of Bone

**Published:** 1847-10-01

**Authors:** 


### Théorie Expérimentale de la Formation des Os. Par *P. Flourens*, Secrétaire Perpetuel de l'Academie Royale des Sciences (Institut de France), &c. Paris, 1847.

### An Experimental Theory concerning the Formation of Bone. By *P. Flourens*, Perpetual Secretary of the Institute of France.

The process of ossification is interesting to the physiologist on several accounts, independently of its bearing on the mere formation of bone. In consequence partly of the mechanical texture, partly of the chemical nature, and partly of the microscopic texure, some of the most important points connected with the first formation and subsequent growth of organic structures, have been made out by the careful investigation of the ossifying process. The activity of deposition going on in growing animals in general may, for example, be inferred from the fact, that the whole skeleton of a young pigeon becomes deeply tinged in twenty-four hours, by giving the animal one meal in which madder has been mixed: by modifying this experiment—by feeding animals with madder, by withholding that substance, and again administering it, some of the interesting phenomena connected with absorption, can be advantageously studied, whilst at the same time light is thrown upon that subtle molecular action, which is incessantly proceeding in the animal economy: again, by tracing, with the aid of the microscope, the various and successive changes displayed in the soft amorphous matter or cartilage, and in the organic cells imbedded in this as in a matrix, and by observing the relations of both these elementary matters to the blood-vessels, several important facts relative to the actions of cells have been determined.

In the treatise before us, among other matters, M. Flourens gives the results of his experimental inquiries respecting a subject of much interest to the practical surgeon—the process, namely, adopted by Nature in the formation of bone. He also re-publishes his experiments upon the effects of madder on the colouration of bone; and concludes with some remarks on what is here called "the great and new problem concerning the relation of forces with matter in living bodies." In the first chapter the author explains his theory on the formation of bone, as embracing the six following propositions:—" 1. That bone is formed in the periosteum. 2. That it increases in thickness by superposed layers. 3. That it increases in length by layers in juxta-position (*par couches juxta-posées.*) 4. That the medullary canal enlarges by the absorption of the internal layers of the bone. 5. That the heads of bones are successively formed and re-absorbed, in order to be again re-formed, so long as the bone grows. 6. That the *continual mutation* of matter is the great and marvellous spring (until now unknown) of the development of the bones." P. 1.

We need not remind our readers that this theory is essentially that of Duhamel, who, in his celebrated and admirable memoirs, contended, to use his own words, that the bones increase in thickness by the superaddition of the layers of the periosteum, which, by becoming ossified, form the substance of the parietes of the medullary canal. We cannot agree with M. Flourens that these views, opposed, as it is well known, by Haller, have been rejected by almost all physiologists. On the contrary, many excellent observers, among whom it will suffice to mention Dupuytren in France, and Howship in this country, have ascertained, in a way that has been deemed by many subsequent writers perfectly satisfactory, that in the reparation of fractured bones, the periosteum does undergo the same changes as those discovered by Duhamel, although the process by which union is ultimately effected is differently described. The subject, however, required further investigation; and we are quite ready to recognise the value of M. Flourens' researches.

In order indisputably to prove the immediate agency of the periosteum in this process, the author excised in several dogs a portion of the rib, (which bone being fixed to the spine and sternum, the divided ends could not fall together), carefully leaving the periosteum. On examining the part four days afterwards, this membrane was found very much swollen and thickened; in six days the periosteum, left between the divided ends of the rib, was already converted into cartilage, and in the midst of this, "two small osseous nuclei were found perfectly distinct, circumscribed, and far distant from the two extremities of the rib;" in sixteen days the nuclei were blended with one, and in three months with both ends of the divided rib. In a subsequent chapter, an account is given of some experiments in which the extremities of the humerus, radius, &c. were removed in young dogs, and in which, the periosteum having been left, the membrane became thickened, osseous nuclei formed in it, and at length in this way the head of the bone was re-produced. From all these researches it is concluded, that the new bone is produced in the periosteum, without contact in the beginning with the old bone; that the original bone does not elongate itself; that its extremities never bud out or rejoin themselves, the new bone being always interposed between them; and, lastly, that the new bone is formed in the periosteum itself, and not in any substance or extravasation whatever foreign to that membrane.

In another set of experiments it is shown that, wherever there is periosteum, bone will be formed; for instance, if a hole be bored in a bone and a canula be introduced into it, the periosteum soon enters the canula, becomes there thickened, cartilaginous, and at last is converted into bone.—(*L. c.*, p. 11.)

In order to establish the second proposition—that the bone, namely, grows by superposed layers, the author surrounded with a ring of platina wire, various bones of dogs, rabbits, Guinea-pigs, &c. "At the end of some time the ring, which at first immediately surrounded the bone, is found surrounded by the bone, and is contained, at length, in the medullary canal;" that is to say, the wire, placed at first between the periosteum and the bare surface of the bone, becomes by degrees covered by new layers deposited externally to it. As regards the increase in length, several experiments are related, like those which Hunter performed for showing the same fact, in which two holes having been made in the tibia of a growing rabbit, it was found, when the animal was killed some time afterwards, that the distance between the holes remained precisely the same, although the bone had become considerably lengthened. This shows, therefore, that the bone does not grow in length by the extension of its tissue, but by the addition of new layers, placed in juxta-position, and formed by the fibro-cartilage, which separates the epiphysis from the shaft.

It has been stated above, that if a wire ring be placed around a growing bone, that at length it is found in the medullary canal. Two explanations have been given of this fact: according to Duhamel the bone is extended, and being pressed by the ring, is broken, in order to be re-united beyond the ring; according to Hunter, on the contrary, it happens that whilst the bone on the one hand acquires external layers which cover the ring, it loses on the other its internal layers which are re-absorbed. In order to determine this point, and also to obviate the objection that a metallic ring, by its pressure and resistance, might break the bone, M. Flourens employed a very small and thin plate of platina, which being isolated and free, could offer no resistance. The result, however, was just the same; the plate became covered with new layers of bone, and the original bone being absorbed, in 36 days the piece of platina was found in the medullary canal. In this manner the fourth proposition is demonstrated—the medullary canal enlarges by the absorption of the internal layers of the bone. The evidence (p. 29) by which the author wishes to prove that, so long as the bones are growing the heads are successively formed and re-absorbed, does not appear to us very satisfactory.

The first chapter concludes with the affirmation of a principle, which, although it is more susceptible, for the reasons already stated, of demonstration in the osseous tissue than elsewhere, is of universal application in animal bodies; it is that of the continual mutation of organic matter. This is proved, in the instance before us, by the experiments made with metallic rings and plates just noticed, which show that the body of the entire bone is without cessation re-absorbed and reformed—"that the continual mutation of matter is the great and marvellous source of the development of the bones." This continual renovation is during growth so very active, that a few weeks suffice in the dog and rabbit for the entire renewal of the shaft of such a bone as the tibia; the longest experiment of this kind lasted only 36 days.

In the succeeding chapter M. Flourens relates some very interesting experiments, illustrative of the powers of absorption and reproduction possessed by the periosteum and the medullary membrane, the ultimate object being to prove *the identity of these two membranes.* It is, however, necessary to explain that what is here called the medullary membrane, is quite distinct from the tissue which lodges the marrow. The latter membrane, it is well known, is nothing else than a portion of the common adipose tissue, and consists, like that, of vesicles in which the medullary matter is contained. The structure and exact relation of the former membrane are most important, and bear so immediately on the present question, that they require to be briefly explained. This so-called medullary membrane is a very thin, and highly vascular cellular tissue, which immediately adheres to the osseous substance, lining firstly the large internal cavity, and from thence being prolonged into all the cells of the cancellated substance and into the Haversian canals, where it meets, and is anatomically confounded with, the processes derived from the periosteum: to this important membrane may be very appropriately applied the term suggested by Dr. Walshe, *endosteum.*

The first position sought to be established is, that "the medullary membrane (endosteum) is the organ which absorbs the internal layers of the bone." We know from the valuable researches of Troja, that if a long bone be sawn across and a stylet be introduced into the medullary tissue, so as to destroy it, necrosis ensues, and a new bone is formed around that which has died. The author desiring to operate on the entire bone, instead of a part only, modified this experiment by making a perforation in the radius of a goat, and then, by the introduction of a stylet into the medullary membrane, destroying the whole of the internal membrane.

In a short time an entirely new radius was formed around the original bone, and with it also a new medullary membrane. But, and this was the immediate object of the experiment, it was likewise formed on examining the external surface of the original and now dead bone, that it was rough and, as it were, eaten into; whilst at the ends of the shaft, the absorption had proceeded so far, that portions of it were completely removed. A careful inspection showed that the agent of this removal was evidently the newly-formed medullary membrane belonging to the new case of bone, and which presented on its internal surface an unequal aspect arising from small mammary eminences with depressions between them. It was found by another experiment, that if the small rib of a rabbit were introduced into the medullary canal of the tibia of a dog, that, after a time, it became corroded and partially absorbed by the thickened medullary membrane.

The following are the author's conclusions from this set of experiments:—"1. That the destruction of the medullary membrane of a bone is followed at first by the death of this bone, and afterwards by the formation of a new medullary membrane and a new bone. 2. That the new bone forms itself in the periosteum of the old bone. 3. That this same periosteum of the old bone produces the new medullary membrane, which at first holds to this periosteum, and only separates from it by the interposition of the new bone. 4. That the internal surface of the new medullary membrane, alternately excavated and mammillated, dissolves and eats away by degrees the old bone, and finishes by absorbing it." P. 41.

By other experiments, in which the periosteum was destroyed, and the bone consequently deprived of its vitality, it was found that the medullary membrane, which had been left intact, had the power of forming a new bone; this became covered by a new periosteum, and was situated in the medullary cavity of the original bone: hence the author concludes that "the medullary membrane does, like the periosteum, produce bone." By these same experiments it was further shown that the periosteum has the power of absorbing bone. Other researches prove, lastly, that the periosteum is capable of producing or forming the medullary membrane.

In considering these investigations, we need scarcely point out how intimately they are connected with the phenomena of necrosis, upon which they throw much additional light. The formation of the osseous case around the enclosed dead bone; the separation of the latter by the absorbing powers of the newly-formed medullary membrane; and the worm-eaten and furrowed appearance of the surface of the sequestrum, are all readily comprehended by the preceding experiments, which are themselves explained by the normal relations of the endosteum and periosteum.

The author investigates in the third chapter a subject which has been a frequent object of experiment—the formation of callus. As it is now generally agreed that the theory of the celebrated Duhamel is in the main correct—that is to say, that the periosteum is the agent by which the new osseous matter is produced—it will suffice, if we merely extract the summary of M. Flourens' researches. "When a bone is fractured, the periosteum begins to swell and thicken, and to send prolongations between the ends of the broken bone; this is, for the periosteum, the first stage; in the second stage, the membrane attaches itself to these fractured extremities, and unites itself to the medullary membrane; then one or several osseous nuclei appear in the periosteum; lastly, these osseous nuclei develop themselves, extend, attach themselves to each end of the broken bone; and the fracture is re-united. Thus all takes place in the periosteum—it is the periosteum which swells; it is the periosteum which attaches itself to the extremities of the broken bone; it is the periosteum which unites itself to the medullary membrane; it is in the periosteum that the osseous nuclei arise and develop themselves; and these nuclei are the callus, the only callus, the intermediate solid which re-unites the fracture, which rejoins the ends of the broken bone." P. 61.

As it is the intention of M. Flourens speedily to publish his microscopic researches on the effect produced by madder on the intimate texture of bone, a subject which has already given in the hands of Mr. Jones and others some very interesting results, we shall not on this occasion notice the account given in the present work, of the general effects observed when bones thus coloured are examined by the naked eye. In the interim, however, we would advise those of our readers who have access to the treatise before us, to peruse the chapters relating to the action of madder, which are illustrated by some beautifully-executed figures.

In the concluding part of this Experimental Theory, the author briefly touches upon one of the most mysterious phenomena of the animal frame—the incessant mutation of organic matter. To the common observer the solid material of the body seems to be fixed and unchangeable; but the philosopher regards it as being continually in a state of motion and renovation, a condition thus happily expressed by Buffon. "That which is, says this illustrious writer, the most constant, the most unalterable in nature, is the print or the mould of each species; that which is the most variable and the most corruptible, is the matter which composes it." The same idea is thus eloquently developed by Cuvier: "In living bodies no molecules remain in their place; all enter and go out successively; life is a continual vortex, of which the direction, all complex as it is, remains constant, as well as the kind of molecules propelled by it, but not so the individual molecules themselves; on the contrary, the actual matter of a living body will soon be there no longer, and yet it is the depository of the forces which will constrain future matter to march in the same direction with itself. Hence the form of these bodies is more essential to them than their matter, since this changes without cessation, whilst the former persists unaltered. It is then a false idea of life to consider it as a simple bond which holds together the elements of the living body; since it is, on the contrary, a spring which moves and transports them incessantly."

Animal chemistry has in some sense, by analysing the débris of the body, given us the measure of this eternal movement. Thus Lehmann has ascertained that, by substituting violent for moderate exercise, the quantity of urea, which represents more especially the waste of the muscular organs, is increased about one-third; whilst Prout, by showing that the proportion of alkaline phosphates in the urine is augmented by mental labour, has proved that a similar law prevails in the nervous system. That the same principle applies to the bones, though in a less degree, is certain; and one of the merits of M. Flourens' researches, is the clear demonstration they afford of this fact, already known from other sources. "The mechanism of the development of the bones is the *renovation,* the *continual mutation* of all the parts which compose them. This bone which I regard, and which grows, has no longer at this moment, any of the matter it had some time since; and soon, it will have nothing of what it now possesses; and yet, in all this perpetual renewal of matter, how little does it change its form."

Having completed our brief review of these Experimental Inquiries, it only remains for us to express the gratification and instruction we have derived from them; a sentiment which we are assured will be re-echoed by all who occupy themselves with the study of physiology.

